# Long-term Opioids Linked to Hypogonadism and the Role of Testosterone Supplementation Therapy

**DOI:** 10.7759/cureus.10813

**Published:** 2020-10-05

**Authors:** Suganya Marudhai, Mauli Patel, Sharathshiva Valaiyaduppu Subas, Mohammad R Ghani, Vishal Busa, Ahmed Dardeir, Ivan Cancarevic

**Affiliations:** 1 Internal Medicine, California Institute of Behavioral Neurosciences & Psychology, Fairfield, USA; 2 Neurology, California Institute of Behavioral Neurosciences & Psychology, Fairfield, USA; 3 Internal Medicine/Family Medicine, California Institute of Behavioral Neurosciences & Psychology, Fairfield, USA

**Keywords:** testosterone, chronic pain, hypogonadism, narcotics, opioids, opioid-induced hypogonadism, testosterone replacement therapy

## Abstract

Opioids play a pivotal role in managing chronic pain with increasing prescription rates over the last few years. Hence, it is crucial to focus on the adverse effects of narcotics, and one of the lesser-known side effects is hypogonadism. Opioids act on the hypothalamus, pituitary, and directly on the gonads affecting serum testosterone levels. Narcotic-induced androgen insufficiency contributes to sexual dysfunction, infertility, hyperalgesia, and involving various body functions overall, affecting the quality of life. Opioid-induced hypogonadism is very challenging to diagnose for the clinicians, as the patients often under-report the symptoms. There are no established guidelines to analyze androgen insufficiency and dealing with their manifestations successfully. We did a substantial search in PubMed and Google Scholar, using various combinations of keywords to collect data to evaluate the impacts of opioids on serum testosterone levels. This study aims to highlight the clinical significance of opioid-induced androgen deficiency and the diagnostic techniques to recognize and credible treatment alternatives, including testosterone replacement therapy. Health care providers should screen the patients routinely for the signs and symptoms and monitor them often for the hormonal changes to select the patients cautiously for testosterone replacement therapy.

## Introduction and background

Recent evidence suggests that as many as 39 million (12% of the population) people in the United States of America (USA) have chronic pain [1]. Opioids have been commonly prescribed for both acute and chronic pain over the past 15 years in the USA [2]. They play an essential role in the disappointment of other non-opioid analgesics, either alone or in combination [3]. As narcotics are the mainstay of pharmacotherapy in chronic pain and palliative therapy, there is an increase in drug-related complications, significantly affecting the patients' quality of life [4]. It is essential to follow specific standard guidelines before prescribing narcotics to forestall overtreatment and to monitor them continuously [4]. Before considering opioids as an option, it is imperative to comprehend the risks and benefits of opioid therapy. 

Narcotics-related adverse effects are receptor-mediated and hence inseparable. Factors like sex, race, and age can also impact the development of side effects. The risk is more noteworthy with higher doses, yet even with lower doses, side effects can still happen on patients who take it for more than 30 days [5]. Short-term opioids-related adverse effects mostly involve the gastrointestinal and the central nervous system, producing nausea, vomiting, constipation, sedation, and respiratory depression. Long-term adverse effects are addiction, dependence, and tolerance. A lesser-known yet one of the common side effects is narcotics suppressing the gonadal function in both genders ranging from 21% to 86% [6]. Opioid-induced hypogonadism is often under-reported and, consequently, underdiagnosed because of low clinician awareness [6]. The impact on the hypothalamic-pituitary-gonadal axis is immediate, and the hormonal changes are dose-related [7]. The most widely recognized symptom of narcotic-induced androgen deficiency is sexual dysfunction affecting 76% of males and 64% of females among long-term opioids users [8]. In males, androgen deficiency leads to sexual dysfunction, fatigue, hot flushes, and night sweats. In females, lower levels of estradiol and progesterone lead to oligomenorrhea, amenorrhea, anovulation, and infertility [9]. Narcotics can reduce the serum testosterone levels by acting on the hypothalamus-pituitary axis, thereby suppressing the release of gonadotrophins [10]. Low testosterone levels can affect pain control leading to hyperalgesia, mood impairment, fatigue, and depression. Opioids have a significant impact on bones affecting bone density leading to osteoporosis, which is a metabolic sequela of hypogonadism [11].

Few management alternatives have been attempted to treat the side effects, including reducing the dose of opioids, discontinuing the opioids, opioid rotation, symptomatic management, switching the route of administration, and considering other pain relief options. In patients who develop hypogonadism symptoms, supplementation with testosterone improves sexual dysfunction and enhances pain sensitivity and quality of life. However, the effectiveness of these strategies is not yet apparent, and we need additional imminent prospective trials and further research to determine the best treatment strategy [12, 13].

From this narrative review, we aim to summarize the underlying mechanism by which opioids affect the testosterone levels, discuss their clinical significance, compare the results from previous studies, and finally make an inference on whether testosterone supplementation can improve the symptoms. We additionally attempted to give clinical guidelines to analyze and prescribe a treatment convention to reestablish testosterone's typical physiological levels. This study will likewise assist with making awareness among the physicians, which will prompt better guiding of the patients.

## Review

Methods

We did an extensive data search from PubMed and Google Scholar using various combinations of the medical subject headings (MeSH) keywords and regular keywords "opioids", "chronic pain", "opioid-induced hypogonadism", "testosterone replacement therapy". We limited the search to include the studies published in English, with available full articles and conducted on humans. We excluded animal studies, non-English studies, case reports, and editorials. We analyzed all the relevant studies to yield more data, applying no time limitations. We aimed our search at identifying the data which focused on the pathophysiology of opioids, the diagnostic tests, and testosterone supplementation.

Discussion

Opioids' Effects on the Hypothalamic-pituitary-gonadal (HPG) Axis

In normal physiological status, the pulsatile release of gonadotrophin-releasing hormone (GnRH) from the hypothalamus stimulates the pituitary to release luteinizing hormone (LH) and follicle-stimulating hormone (FSH). LH acts on the testes (Leydig cells) and ovaries (theca cells) to produce testosterone [14]. Testosterone is aromatized to estradiol, which is also derived through the peripheral conversion of oestrone. Oestrone is secreted directly from the adrenal glands and also through the aromatization of androstenedione, a product of testosterone [15]. FSH acts on Sertoli cells to produce sex hormone-binding globulin (SHBG) and inhibin. FSH also supports the production and maturation of sperm cells and stimulates the development of ovarian follicles. The sex hormones and inhibin have a negative feedback mechanism on LH, FSH, and GnRH [14].

**Figure 1:**
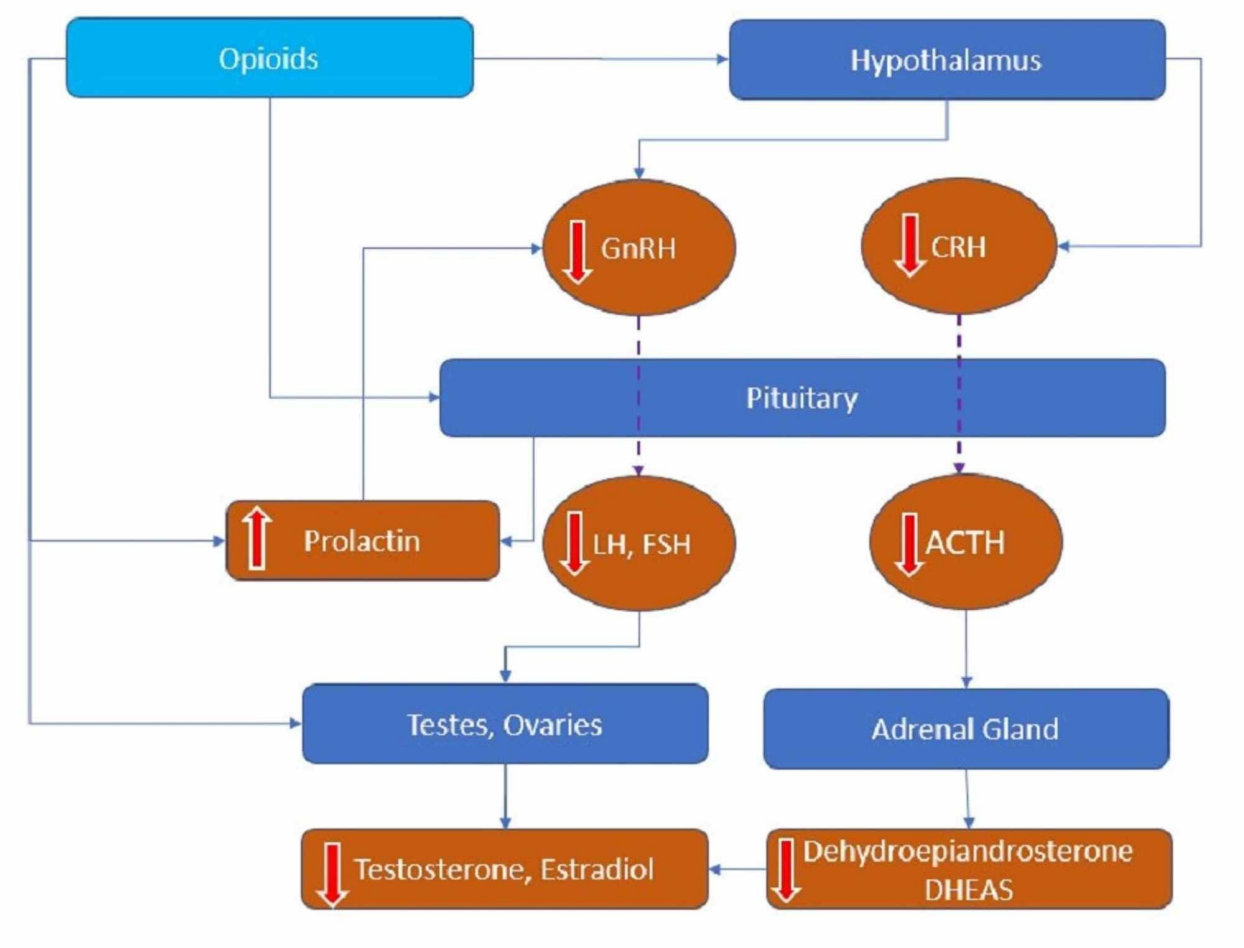
Opioids effects on the hypothalamic-pituitary-gonadal (HPG) axis GnRH: gonadotropin-releasing hormone; CRH: corticotropin-releasing hormone; LH: luteinizing hormone; FSH: follicle-stimulating hormone; ACTH: adrenocorticotropic hormone; DHEAS: dehydroepiandrosterone sulfate

Narcotics act directly on the hypothalamus through its receptors guanine nucleotide-binding protein (G-protein)-coupled mu-opioid peptide (MOP) receptors and inhibit the pulsatile release of GnRH, which blocks the release of LH and FSH from the pituitary, affecting the production of testosterone or estrogen from the gonads. Moreover, narcotics also act directly on the pituitary gland to suppress the release of gonadotropins [16]. They also increase prolactin levels, which can inhibit the secretion of GnRH and cause low testosterone levels [17]. Ragni et al. showed that there was significantly lower LH, FSH, testosterone, and high prolactin levels (12.3 +/- 10.5 vs. 5.9 +/-2.5 ng/ml, P < 0.025) in chronic heroin users when compared to the control group, with normal radiography of the sella turcica even with prolactin levels > 15 ng/ml [18]. Their study also implied that hyperprolactinemia is not associated with any particular type of opioid but can be related to environmental stress factors and the social conditions seen in heroin addicts. Prolactin suppresses the pulsatile release of GnRH and acts directly on the pituitary affecting LH secretion, leading to lower levels of sex hormones [17]. Patients with high prolactin present with galactorrhea, painful gynecomastia, infertility, and irregularities with menstrual cycles. Opioids negatively affect the release of corticotrophin-releasing hormone (CRH) and vasopressin at the hypothalamus. Low CRH down-regulates the adrenocorticotrophic hormone (ACTH) secretion leading to decreased levels of dehydroepiandrosterone and dehydroepiandrosterone sulfate (DHEAS) [19]. DHEAS is a sensitive marker of opioid-induced adrenal deficiency, the precursor of testosterone in the adrenal gland [20]. Lower levels of dehydroepiandrosterone sulfate (DHEAS) can add to fatigue, weakness, depression, and sexual dysfunction. Rhodin et al. have noted lower levels of DHEAS (1.56 mol/L vs. 2.71 mol/L; P < 0.05) and higher levels of prolactin (25.1 g/L vs. 8.88 g/L) (P < 0.001 ) in females on opioids when compared to the controls [19]. Reduced levels of DHEAS in female patients on opioids clearly explain hypoadrenalism caused by opioids, which leads to low adrenal androgen production [20]. DHEAS replacement therapy helps in improving sexual dysfunction and fatigue in patients with low DHEAS levels. Opioids can reduce the production of sperm, testicular interstitial fluid, and testosterone by acting directly on the gonads through the MOP receptors. It also has some detrimental effects on semen parameters like affecting the sperm count (oligozoospermia), morphology (teratozoospermia), forward motility (asthenospermia), semen volume (hypospermia), and sperm concentration [18]. In the study population, asthenospermia was reported in all cases of opioid users, and hypospermia and teratozoospermia to a lesser extent [18]. In chronic opioid users, when compared to healthy adults, a reduction in the sperm concentration (22 million vs. 66 million/dl; P = 0.004), reduced activity of superoxide dismutase and catalase, and an increase in the fragmentation of deoxyribonucleic acid (DNA) (36% vs. 27%) were noted [21]. The study proved the association between the opioid doses and impaired semen parameters with the group on high doses had a high percentage of sperm DNA damage and diminished semen parameters. Narcotics have a significant effect on semen parameters like quality, antioxidant capacity, sperm function, and DNA integrity. Opioid-induced hypogonadism is both through its central and peripheral effects, as summarized in Figure 1 below.

According to Roberts et al., after administering intrathecal opioids, the testosterone levels reduced significantly from 7.7 +/- 1.1 nmol/I at baseline to 2.0 +/- 0.7, 2.8 +/- 0.5, 4.0 +/- 0.9 nmol/L at 1, 4, and 12 weeks, respectively (P < 0.0001) [22]. LH and FSH were in the normal physiological range and not increased, explaining central hypogonadism. This study explains the suppression of the HPG axis with opioids through its receptors, without an increase in LH describing a central effect. Daniell demonstrated that among men on oral opioids, 89% showed reduced levels of free testosterone, dihydrotestosterone, estradiol, LH, and FSH (P < 0.0001), and 87% reported erectile dysfunction and reduced libido [23]. Daniell also proved that in opioid-consuming postmenopausal women, independent of BMI and age, average values of LH and FSH were 70% to 73% lower than the control group (P < 0.001), with median values of 4.1 and 32.5 mIU/ml for LH and 7.1 and 71.0 mIU/ml for FSH [24]. Rhodin et al. found in their study on female patients on opioids that the mean estradiol value was 208 pmol/L vs. 510 pmol/L (P < 0.05), DHEAS levels were 40.6 vs. 65.3 mg/l, the LH peak was 17.6 IE/L vs. 38.3 IE/L (P < 0.01), and the baseline FSH was 25.7 IE/L vs. 60.7 IE/L (P < 0.05) compared to the controls [19].The data supports that opioid-induced hypogonadism is through the suppression of the HPG axis along with ovarian and adrenal androgen production explaining both central and peripheral effects.

Moreover, the effect of opioids on the HPG axis gets accentuated due to various other factors like age, chronic pain, comorbidities like anxiety, and drug therapies like chemotherapy, which can influence the hormonal levels [25]. Hence the diagnosis of opioid-induced hypogonadism is very challenging. There are guidelines to initially screen the patients on opioids with ADAM (androgen deficiency in aging males) questionnaire, and if this is positive, then follow up with measuring serum testosterone levels is recommended [26]. Once the diagnosis is suspected, we need to measure total testosterone, free testosterone, estradiol, LH, FSH, sex hormone-binding globulin, DHEAS, an evaluation of adrenal function, and always collaborate the care with an endocrinologist to confirm the diagnosis. There are no particular guidelines or recommendations to diagnose hypogonadism in women other than measuring estradiol in addition to testosterone, LH, FSH, DHEAS, and bone density to guide treatment [27].

Clinical Significance of Low Testosterone

Testosterone, more than being considered a sex hormone, has different roles in various body systems' metabolic functions. The hormone's significant anabolic effects include increasing the muscle mass, exercise tolerance, maturation of bone, and linear growth [28]. It has a substantial influence on general health, mood, and wellbeing through its actions on the cognitive centers in the brain [29]. Various factors like aging, lack of physical activity, excess distribution of body fat tissue, smoking, and even medical conditions like diabetes mellitus, hepatic dysfunction, hypothyroidism, obesity, serum albumin, and sex hormone-binding globulin (SHBG) levels can influence the level of testosterone [30]. Low testosterone levels are associated with dyslipidemia, weight gain, glucose intolerance, poor tissue healing, mood changes, depression, fatigue, anxiety in both sexes [31]. Low testosterone levels are associated with osteopenia and osteoporosis and increase the risk of osteoporotic fractures by 50-60% [32]. Opioids affect bone metabolism by directly acting on the osteoblasts and indirectly through the suppression of testosterone, which reduces bone mineral density leading to osteoporosis. Few studies prove the association of low androgen levels with a high incidence of coronary heart diseases (CHD) and reveal that serum testosterone levels are inversely related to deaths with CHD [33]. The relationship between the low testosterone levels and CHD can be explained by the fact that it can influence serum high-density lipoprotein (HDL), low-density lipoprotein (LDL), and total cholesterol levels [34].

The prevalence of hypogonadism is significantly different between men and women, depending on the route of opioid therapy. With long-term oral opioids, women have a lower prevalence than men, but with intrathecal opioids, the prevalence of androgen deficiency is higher in both genders [24]. In a study among chronic pain patients using intrathecal opioids, reduced libido and potency were reported in 96% of men with lower levels of testosterone and free androgen index (FAI = testosterone/SHBG) (P < 0.001), and 69% of women presented with decreased libido, irregularities in the menstrual cycles, and anovulation with lower levels of LH, FSH, estrogen, and progesterone [9]. In women, long-term opioids affect the hormonal levels of LH, FSH, testosterone, and progesterone, which has a significant impact on their menstrual cycles [16]. In addition to irregularities in their menstrual cycles, the inhibition of adrenal androgens and ovarian sex hormones can also cause anovulation, reduced fertility, depression, and osteoporosis [16]. Daniell has reported that among women on long-term intrathecal opioids, 70% had amenorrhea (P < 0.05), 30% presented with irregularities in the menstrual cycles, and 84% reported sexual dysfunction along with fatigue, depression, and increased risk of osteoporotic fractures before age 60 (P < 0.05) [24].In men, the most significant symptoms are erectile dysfunction, reduced ejaculatory volume, problems with achieving orgasm, decreased libido, and infertility. Still, fatigue, depression, less body hair, breast pain, gynecomastia, shrinking testes, reduced muscle mass, low concentration, mood disturbance, night sweats, and hot flushes can also be noted [35]. Opioids can lead to the endocrine changes immediately as early as four hours, and it is easily reversible once discontinued within 24 hours [7, 23]. Patients on higher doses of narcotics have an increased impact on testosterone levels. For an increase of 10 mg in the methadone dose, a reduction of 0.97 ng/dl (P = 0.003) in the testosterone levels was noted [36]. The study explains the inverse relationship between the methadone dose and the testosterone levels (CI -0.003, -0.000, P < 0.018) [36]. Data suggests that when the daily morphine dose exceeds 100-200 mg equivalents, we could expect significant effects on the endocrine changes [37]. Long-acting opioids have a significant association with hypogonadism as they cause GnRH suppression. Rubinstein et al. proved that compared to men on short-acting opioids (SAO) (34%), men on long-acting opioids (LAO) (74%) after controlling for the BMI and daily dose, showed a significant rate of hypogonadism (odds ratio of 4.78, 95% CI 1.51-1.5.07, P = 0.008) [7]. Both LAO and SAO can cause the acute suppression of GnRH when they reach the threshold serum levels. The more significant suppression of GnRH with LAO is due to the fact that LAO achieve more stable serum levels, whereas SAO varies throughout the day [7]. When opioid-induced hypogonadism is suspected, obtaining a thorough history before measuring the serum testosterone levels is of paramount importance. Testosterone levels peak in men in the mornings but fluctuate during the daytime; hence the diagnosis should be based on multiple measurements than a single reading [38].

Role of Testosterone Supplementation

Narcotic-induced androgen deficiency should be managed initially with conservative measures like diet and lifestyle modifications. When these traditional measures fail, consider other options like reducing the dose, switching to a different opioid, or switching to a non-opioid analgesics like non-steroidal anti-inflammatory drugs (NSAIDs) and the hormonal replacement therapy. Patients develop the classic symptoms of hypogonadism with testosterone levels lower than 300 ng/dl [39]. However, in most cases, the serum androgen levels are variable; therefore, evaluating the patients based on their signs and symptoms is essential. Hence, we should diagnose the patients based on both the clinical assessment of symptoms and the laboratory parameters [40]. It is even more critical to decide when to start the androgen supplementation therapy and who can get benefit from such treatment. When considering the testosterone replacement therapy, careful attention to exclude the patients with breast or prostate cancer, high prostate-specific antigen (PSA) levels, hematocrit > 50%, untreated obstructive sleep apnea, and poorly controlled heart failure [40]. Moreover, the risks of hypercalcemia, polycythemia, and lipid abnormalities should also be considered [37]. The target range of testosterone levels is between 400-700 ng/dl for men and women 20-80 ng/dl [40]. Each laboratory will have its cut-off values, but a range of 280-800 ng/dl is recommended [39].

The main aim of replacement therapy is to maintain the normal serum levels of testosterone between the intervals of administration. For women, hormonal replacement therapy (HRT) may include the administration of estrogen and progestin or oral contraceptives to improve the symptoms. DHEAS (50 mg daily), a precursor of testosterone, can be used as a hormonal supplement, but it is not a standardized therapy [41]. HRT for women hence includes various doses of estrogens and progestins with a significant risk for breast cancer and cardiovascular diseases.

Though various testosterone preparations are available for the replacement therapy in men, it should be chosen based on the cost, patient choice, and the burden of the treatment [40]. Oral preparations are a good option when it is introduced first because of good compliance among patients. However, they have a major risk of hepatotoxicity due to its first-pass metabolism through the liver. The intramuscular preparations (75-100 mg once every week or 150-200 mg every two weeks) are the most cost-effective [42]. But they can make the testosterone reach the supraphysiological levels after administration and then suddenly drop to sub-physiological levels before the next administration [42]. Mood swings, acne, and injection site pain are few reported side effects. It is imperative to monitor the patients with hematocrit as there is a greater risk of polycythemia in the first few months after starting the therapy [42]. Transdermal preparations available in the form of patches and creams are also effective. The most recommended one is 1% hydroalcoholic gel (50-100 mg of testosterone every day), which can dry fast with good bioavailability [40]. In older patients, the gel formulations are the first choice as it can be rapidly reversed if any adverse effects develop. But there is a possibility for secondary transfer through skin-skin contact and can increase the serum testosterone levels. Transdermal patches (delivers 5-10 mg of testosterone over 24 hours) can be applied every night but should be observed for any allergic reaction [40]. Testosterone pellets are available for subcutaneous injections in different doses at an interval of 3-6 months [43]. We should measure the baseline levels of free testosterone, total testosterone, LH, FSH, prolactin, prostate-specific antigen, and complete blood count before starting the therapy. The labs should be repeated after 3-6 months after starting the therapy, then every 3-12 months after that as needed [40]. We should also monitor them with complete blood count, liver function tests, coagulation studies, serum calcium, and lipid profile along with a dual-energy x-ray absorptiometry scanning (repeat after one or two years) during the therapy [40]. Testosterone replacement therapy (TRT) has significant risks of stimulating the growth of the prostate gland, gynecomastia, priapism, male pattern baldness, abuse potential, low HDL (high-density lipoproteins), oligospermia, and azoospermia [40]. In a double-blinded study of 41 men with total testosterone < 12 nmol/l ( testosterone injections three times in six months vs. placebo injections), TRT has improved the median serum total testosterone levels from 6.8 nmol/l (5.0; 9.3) to 12.3 nmol/l (7.0; 19.9) (P < 0.001 vs. placebo), increased lean body mass 3.6 kg (2.3; 5.0) (TRT) vs. 0.1 kg (-2.1; 1.5) (placebo) and lowered the total body fat -1.2 kg (-3.1; 0.7) vs. 1.2 kg ( 0.9; 2.5) both P < 0.003, without much improvement in pain perception [44]. There was no significant change with pain perception, though TRT has shown improvement in body composition like improving the lean body mass and lowering the body fat.

Low testosterone is associated with increased pain sensitivity as it interferes with opioid analgesia, and it can be reversed with testosterone supplementation therapy (TST). A retrospective pilot analysis examined the effect of testosterone supplementation therapy on chronic pain based on the Numerical Rating Scale (NRS), daily morphine (MED) equivalent dose, and the impact of TST on the hypogonadal symptoms measured with the International Index of Erectile Function (IIEF-5) and Androgen Deficiency in Aging Males (ADAM) [45]. Raheem et al. have shown that there was an increase in the median testosterone by 262.5 ng/dl (P < 0.05) with an improvement of hypogonadal symptoms (ADAM and IIEF-5 scores) like improving the sexual desire, erectile function, body composition, and quality of life (P < 0.05) in TST group [45]. The data also proves the improvement in the Numerical Pain Rating Scale (NRS) (P = 0.02) and reduction in the daily morphine equivalent (MED) (P < 0.05) in testosterone supplemental therapy (TST) group vs. non-TST group [45]. TST has a significant role in reducing the opioid dose requirement and improving the pain perception and hypogonadal symptoms. In a randomized study of 53 female patients with acquired immunodeficiency syndrome (AIDS) wasting syndrome with low serum androgen levels, transdermal testosterone improved the serum testosterone levels in a dose-dependent manner (P < 0.0001), caused weight gain ( amounting to 4% increase of initial weight) (P = 0.041) and increased the fat-free mass to a greater extent, improved the quality of life in terms of general physical health, emotional wellbeing, energy and social function (P= 0.024) and improved the pain score evaluated based on the pain item on 36 items short-form health survey (SF-36) (P= 0.059) [46]. These studies have demonstrated that TST has improved the pain perception and serum androgen levels along with significant improvement in the quality of life in terms of improving the energy level, physical and mental wellbeing. In this comprehensive literature review, we did not include any methodological quality assessment criteria. As well, we did not assess a specified population group based on their age or ethnicity.

## Conclusions

Opioid-induced hypogonadism is a lesser-known but highly prevalent adverse effect in patients on long-term opioid therapy. Narcotics have both central and peripheral effects causing reduced serum testosterone levels. The clinicians should look for these lesser known adverse consequences and assess them clinically based on their signs and symptoms. Testosterone replacement therapy is a viable option for managing symptomatic males, and we recommend collaborative care with an endocrinologist for the best outcome. Careful patient selection and close monitoring during therapy are the prerequisites for a successful therapy. We need further studies to provide us more details on the prevalence of opioid-induced hypogonadism and proper guidelines on diagnosis and treatment.
